# Collimator and Energy Window Evaluation in Ga-67 Imaging by Monte Carlo Simulation

**DOI:** 10.4274/mirt.galenos.2020.21549

**Published:** 2020-10-19

**Authors:** Mina Ouahman, Rachid Errifai, Hicham Asmi, Youssef Bouzekraoui, Sanae Douama, Farida Bentayeb, Faustino Bonutti

**Affiliations:** 1 Mohammed V-Rabat University Faculty of Science, Laboratory of High Energy Physics Modelisation Simulation, Rabat, Morocco; 2 Academic Hospital of Udine, Clinic of Medical Physics, Udine, Italy

**Keywords:** Ga-67 imaging, primary photons (original), penetration, scatter, SIMIND, sensitivity

## Abstract

**Objectives:**

Gallium-67 (Ga-67) imaging is affected by collimator penetration and scatter components owing to the high-energy (HE) gamma-ray emissions. The characterization of penetration and scatter distribution is essential for the optimization of low-energy high-resolution (LEHR), medium energy (ME), and HE collimators and for the development of an effective correction technique. We compared the image quality that can be achieved by 3 collimators for different energy windows using the SIMIND Monte Carlo code.

**Methods:**

Simulation experiments were conducted for LEHR, ME, and HE collimators for Ga-67 point source placed at 12-cm distance from the detector surface using the Monte Carlo SIMIND simulation code. Their spectra point spread functions as well as the original, penetration, scattering, and X-rays curves were drawn and analyzed. The parameters full-width at half maximum and full-width at tenth maximum were also investigated.

**Results:**

The original, penetration, and scatter curves within 10% for LEHR were 34.46%, 33.52%, 17.29%, and 14.72%, respectively. Similarly, the original, penetration, scatter, and X-rays within 10% for ME and HE were 83.06%, 10.25%, 6.69%, and 0% and 81.44%, 11.51%, 7.05%, and 0%, respectively. The trade-off between spatial resolution and sensitivity was achieved by using the ME collimator at 185 photopeak of Ga-67.

**Conclusion:**

The Monte Carlo simulation outcomes can be applied for optimal collimator designing and for the development of new correction method in Ga-67 imaging.

## Abstract (tr)

**Amaç:**

Galyum-67 (Ga-67) görüntüleme, yüksek enerjili gama ışını emisyonları nedeniyle kolimatör penetrasyonundan ve saçılma bileşenlerinden etkilenir. Penetrasyon ve saçılma dağılımının karakterizasyonu düşük enerjili yüksek çözünürlüklü (LEHR), orta enerjili (ME) ve yüksek enerjili (HE) kolimatörlerin optimizasyonu ve düzeltme yönteminin geliştirilmesi için önemlidir. Bu çalışma, SIMIND Monte Carlo kodunu kullanarak farklı enerji pencereleri için üç kolimatör ile elde edilebilen görüntü kalitesinin karşılaştırılmasını amaçlamaktadır.

**Yöntem:**

Dedektör yüzeyinden 12 cm uzağa yerleştirilen Ga-67 nokta kaynağı ile LEHR, ME ve HE kolimatörleri için Monte Carlo SIMIND simülasyon kodu kullanılarak simülasyon yapıldı. Spektrumları yayılma fonksiyonlarına işaret ediyordu ve ayrıca orijinal, penetrasyon, saçılma ve X-ışınları eğrileri çizilerek analiz edildi. Yarı maksimumdaki tam genişlik ve 1/10 maksimumdaki tam genişlik araştırıldı.

**Bulgular:**

LEHR için %10 içindeki orijinal, penetrasyon, saçılma ve X-ışınları sırasıyla %34,46, %33,52, %17,29 ve %14,72 idi. ME için %10 içindeki orijinal, penetrasyon, saçılma ve X-ışınları sırasıyla %83,06, %10,25, %6,69 ve %0 idi. HE için %10 içindeki orijinal, penetrasyon, saçılma ve X-ışınları sırasıyla %81,44, %11,51, %7,05 ve %0 idi. Uzamsal çözünürlük ve duyarlılık arasındaki denge, Ga-67’nin 185 fotopikli ME kolimatörü kullanılarak elde edildi.

**Sonuç:**

Monte Carlo simülasyon sonuçları, Ga-67 görüntülemede optimum kolimatör tasarımı ve yeni düzeltme yönteminin geliştirilmesi için kullanılabilir.

## Introduction

Gallium-67 (Ga-67) disintegrates by electron capture to stable zinc 67 with a radioactive half-life of 3.26 days, and it has less costly imaging requirements. Despite that ^18^F-fluorodeoxyglucose positron emission tomography is currently used for the diagnosis of non-Hodgkin’s lymphoma, Ga-67 scintigraphy remains useful during the early period of treatment (1,2,3). The decay scheme of Ga-67 involves multiple emission energies with photopeak energies at 93, 185, and 300 keV. Therefore, the contributions of some photons are included in lower photopeak energy window. In addition, lead X-rays produced in the collimator can also be detected in the 93-keV photopeak energy windows. This contribution degrades the image quality and the quantitative accuracy, especially, when imaging with a low-energy collimator (4,5). All photons detected with the collimator were grouped into 3 categories as follows: original photons (i.e., photons detected on the detector without any scatter or penetration), penetration photons (those that passed through septa without attenuation), and scatter photons (those that scattered at least once in the septa) (6,7). Only the first photons provide correct information. Image quality is essentially affected by the penetration and scatter components of the collimator, particularly in high-energy imaging. The scattered photons depend on the photons energy, object study, and collimator design. Gamma-camera cannot classify the image-forming photons into original, penetrated, or scattered photons. The knowledge of penetration and scatter distribution is essential for the optimization of collimator design and for the development of a correction method (7,8). The typical energy resolution of NaI (Tl) has a full-width at half maximum (FWHM) of approximately 10% at 140 keV. Therefore, the contribution of scatter within the photopeak energy windows is huge (9). Accordingly, several compensation scatter methods have been proposed, for example, the triple-energy window method and the Compton window subtraction method (10,11). Therefore, the Monte Carlo simulation technique (12,13,14,15,16,17,18) separates the original, penetration, and scatter contribution inside the photopeak window. In this study, we compared the simulated energy spectra in Ga-67 imaging for different parallel-hole collimators for the Siemens Symbia gamma-camera (Table 1). The resolution and sensitivity (cps/MBq) were accordingly evaluated. We also estimated and compared the original, penetration, and scatter contribution inside the 20% and 10% energy windows around the 93, 185, and 300 keV photopeaks. Through this work, we aimed to determine the optimal energy window and collimators design [low-energy high-resolution (LEHR), medium energy (ME), and high energy (HE)] in Ga-67 imaging.

## Materials and Methods

No statistical analysis was performed, and the study has no evident limitations.

Our study did not involve any patients.

All procedures performed in the experiments involving human participants were in accordance with the ethical standards of the institutional and/or national research committee and with the 1964 Helsinki Declaration and its later amendments or other comparable ethical standards.

We used the SIMIND Monte Carlo simulation code (version 6.1) to simulate a point source of Ga-67 isotope (Table 2) of dimension 0.1x0.1x0.1 cm^3^ located at the center of a cylindrical water phantom of dimension 16x22x22 cm^3^ and placed at 12 cm from the detector surface. In this simulation, we modeled the Siemens Medical System Symbia equipped by the following 3 collimators: LEHR, ME, and HE (Tables 1, 2) (19). A detector of 59.1x44.5-cm^2^ area and 0.95-cm NaI (Tl) crystal thickness was used.

The detector was characterized by an intrinsic spatial resolution of 0.34 cm and an energy resolution of 8.80% at 140 keV. The photomultiplier tube (PM) back-scatter material with a thickness of 10 cm was used to simulate the backscattering of photons from the light guides and PM.

We included the contribution of lead X-rays scatter photons inside the collimator lead. The aluminum cover material thickness was 0.1 cm. The pixel size in the simulated planar images was 0.34 cm and 128x128 matrix size. We imported the binary image to the ImageJ software created by SIMIND. At the end of each simulation, SIMIND provided the value of original, penetration, and scatter photons as well as the efficiency, sensitivity, FWHM, and full-width at tenth maximum (FWTM) in separate files. Table 3 shows the abundance of Ga-67 as a function of energy, which is extremely useful in the selection of appropriate collimator.

## Results

**Figure 1:**
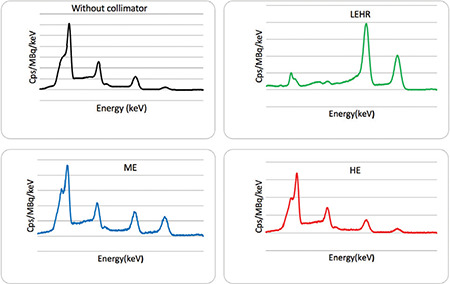
Comparison of the total simulated spectrum among the 3 different collimators for Ga-67 imaging Ga-67: Gallium-67, LEHR: Low-energy high-resolution, ME: Medium energy, HE: High energy

Figure 1 depicts the simulated energy spectra of a Ga-67 point source in water placed at 12-cm distance away from the detector surface. All energy spectra include both scattered and unscattered photons. The energies displayed in Figure 1 represent the energies of the main emission peaks of the isotope. The spectra characteristics help explain the choice of collimator type for imaging. The total number of photons detected with the collimators were degraded widely relative to that without the collimators. The peaks was detected in the energy region of 70-86-keV for each collimator, which matched the characteristic X-rays of lead produced by the photoelectric effect of HE photons. According to the contribution of penetration and scatter components in the projection data, the shape of Compton edge and Compton region were found to be different among the 3 collimators. In addition, as it can be seen, the contribution of septal penetration and scatter in the HE and ME were less than those in the LEHR.

Table 4 depicts the energy windows used for each collimator. The results of the simulation are detailed in Table 5. We evaluated the original, septal penetration, and scatter components in parallel-hole collimators LEHR, ME, and HE for Ga-67 point source using the Monte Carlo simulation program.

**Figure 2:**
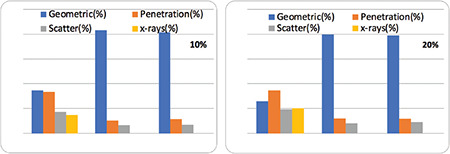
Comparison among the original, penetration, scatter, and X-rays for LEHR, ME, and HE collimators LEHR: Low-energy high-resolution, ME: Medium energy, HE: High energy

Figure 2 illustrates the comparison of the proportion of penetration (photons that penetrated the collimator), scatter (those that scattered in the collimator), and original (those that were detected on the detector without any scatter or penetration) inside the 20% and 10% energy windows around the 93-, 185-, and 300-keV photopeaks in the LEHR, ME, and HE collimators, respectively.

**Figure 3:**
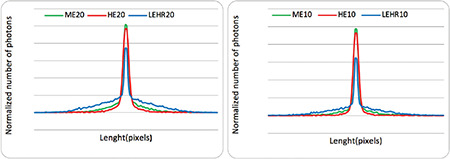
Comparison of PSF among the 3 different collimators for the Ga-67 point source inside the 20% and 10% energy windows with HE, ME, and LEHR collimators PSF: Point spread functions, Ga-67: Gallium-67, LEHR: Low-energy high-resolution, ME: Medium energy, HE: High energy

The point spread functions (PSF) were studied for Ga-67 imaging. A point source of 0.1-mm diameter was acquired with different collimators. The PSF obtained for all collimators are represented in Figure 3. The curves of Ga-67 with the ME and HE collimators demonstrate the effects of the septal that lowers the resolution. LEHR offers a poorer resolution than the HE and ME collimators within 20% and 10% windows (20,21,22) owing to the septal penetration and the scattering effects.

**Figure 4:**
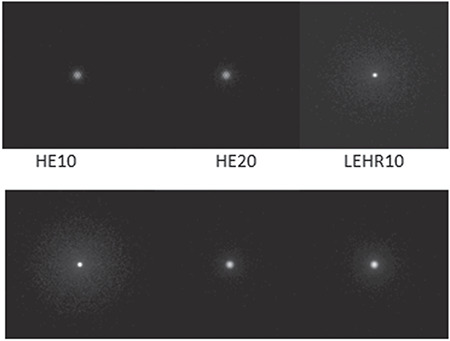
Images of the Ga-67 point sources created at the end of each simulation with LEHR, ME, and HE collimators within the 10% and 20% energy windows Ga-67: Gallium-67, LEHR: Low-energy high-resolution, ME: Medium energy, HE: High energy

The indices of resolution used were FWHM to measure the collimator’s spatial resolution, and the FWTM was used as an index of septal penetration and Compton scattering within the collimator. In order to quantify the resolution, FWHM and FWTM were computed on the PSF. The results for both FWHM and FWTM and the relevant sensitivities (Cps/MBq) are given in Table 6. These parameters were compared within the 20% and 10% energy windows. It can be seen from the table that, for each collimator, the FWTM increased with the width of the energy windows, especially for the LEHR and ME collimators, while, the FWHM remained approximately the same (21,22,23). On the other hand, the sensitivity decreased when the width of the energy window decreased, which was an extremely sharp transition for LEHR. Figure 4 depicts the images of Ga-67 point sources obtained from the simulation performed with different collimators.

## Discussion

The Monte Carlo simulation SIMIND code was used to store the history of the detected events, which was otherwise not possible with the experimental data. However, to compare i.e., equal acquisition time were employed. The collimator-detector response (CDR) of the single photon emission tomography imaging system depends on the following 4 components: the intrinsic response and the response of the detector and the original, penetration, and scatter distribution inside the collimator. Therefore, the characterization of this components helps compensate the CDR, which has a significant effect on accurate quantification (20). As the Ga-67 radionuclide emits multiple-energy rays, a large scattered event is detected within the photopeak energy windows; this event degrades significant contrast and lesion detection (9).

The low original component in LEHR collimator may be attributed to the high level of penetration, scattering, and X-rays effects. This component is large for ME and HE collimators. The original component (primary photons) decreased with an increase in the photopeak window, especially for the LEHR collimator. Penetration and scatter component increased with an increase in the photopeak window, demonstrating smooth increase in all collimators. In addition, the X-rays component showed a slow increase with an increase in the photopeak window in the LEHR collimators. Although the number of detected photons from the main energy peak (93 keV) was high for LEHR, it is important to consider that a large amount from this peak was detected after septal penetration. The indices of resolution used were FWHM to measure the collimator’s spatial resolution, while FWTM served as an index of septal penetration and Compton scattering within the collimator. In order to quantify the resolution, FWHM and FWTM were computed on the PSF.

The presence of a high level of penetrated and scattered photons from the LEHR collimator degrades spatial resolution, contrast, and quantification (20,21,22,23,24). Table 6 depicts that the use of LEHR collimator with Ga-67 imaging resulted in the most degraded spatial resolution, while the use of an HE collimator in Ga-67 imaging resulted in the loss of sensitivity and spatial resolutions (25). Data presented in Table 5 signify that the trade-off between the sensitivity and spatial resolution achieved with the ME collimator occurred when the photopeak was centered over the 185-keV photopeak with the use of 10% photopeak. We hence recommend Ga-67 imaging with a single peak around the 185-keV peak considering the high relative intensity of the 185-keV gamma peak (30%) and the high absorption efficiency of this photopeak within the NaI (Tl) crystal.

The fogginess in these images increased with an increase in the energy window, especially for LEHR. This observation can be attributed to the HE photons detected inside the energy window. Therefore, the LEHR collimators became virtually transparent, which was evident from the calculated value of high septa penetration and scattering obtained from the simulation experiments (Table 5).

## Conclusion

We studied the LEHR, ME, and HE collimator in the Ga-67 imaging. Based on our results, we noted loss in sensitivity and spatial resolution by the HE collimator, as the LEHR collimator allows poorer spatial resolution. On the other hand, the trade-off between resolution and sensitivity was achieved with an ME collimator in the 10% energy window with a single peak at 185 keV. We believe that our results would facilitate the designing of optimal collimator and the development of a new correction method in Ga-67 imaging.

## Figures and Tables

**Table 1:**
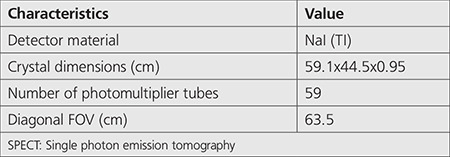
Specifications of the Siemens SYMBIA SPECT scanner

**Table 2:**
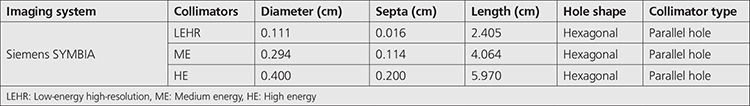
Collimators data of the Siemens SYMBIA systems during simulation

**Table 3:**
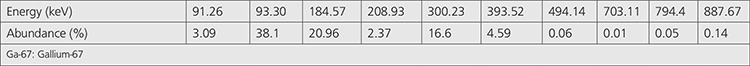
Energies and intensities of gamma rays emitted from the Ga-67 source

**Table 4:**
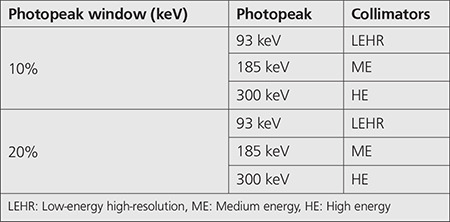
Acquisition energy windows used in the simulation

**Table 5:**
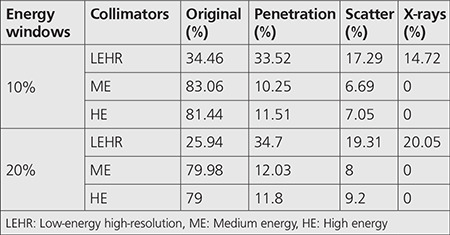
The result of the simulation performed in the study

**Table 6:**
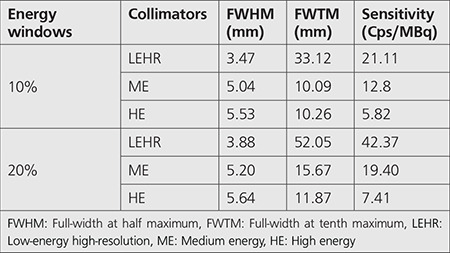
Calculated FWHM and FWTM and sensitivities within the 20% and 10% energy windows for LEHR, ME, and HE collimators

## References

[1] Mansberg R, Wadhwa SS, Mansberg V (1999). Tl-201 and Ga-67 scintigraphy in non-Hodgkin’s lymphoma. Clin Nucl Med.

[2] Tuli MM, Al-Shemmari SH, Ameen RM, Al-Muhanadi S, Al-Huda AF, Ballani N, Khoshi M, Al-Enezi F, Bajciova V, Mottl H (2004). The use of gallium-67 scintigraphy to monitor tumor response rates and predict long-term clinical outcome in patients with lymphoma. Clin. Lymphoma.

[3] Shinohara H, Koga Y (1981). Ga-67 imaging with scintillation camera – The selection of collimator. J Nucl Med.

[4] Farncombe TH, Gifford HC, Narayanan MV, Pretorius PH, Bruyant P, Gennert M, King M (2002). An optimization of reconstruction parameters and investigation into the impact of photon scattering Ga-67 SPECT. IEEE Trans Nucl Sci.

[5] Moore SC, Kijewski MF, Fakhri GEE (2005). Collimator Optimization for Detection and Quantitation Tasks: Application to Gallium-67 Imaging. IEEE Trans Med I.

[6] Vandenberghe ERS, Holen RV, Beenhouwer JD, Staelens S, Lemahieu I (2007). Comparison of image quality of different iodine isotopes (I-123, I-124 and I-131). Cancer Biother Radiopharm.

[7] Dewaraja YK, Ljungberg M, Koral KF (2000). Characterization of Scatter and Penetration Using Monte Carlo Simulation in 131I Imaging. J Nucl Med..

[8] Lewis DP, Tsui BMW, Tocharoenchai C, Frey EC (1998.). Characterization of medium and high energy collimators using ray-tracing and Monte Carlo methods. 1998 IEEE Nucl Sc Symp and Med Imag Conf.

[9] Farncombe TH, Gifford HC, Narayanan MV, Pretorius PH, Frey EC and King MA (2004). Assessment of scatter compensation strategies for Ga-67 SPECT using numerical observers and human LROC studies. J Nucl Med.

[10] Jaszczak RJ, Greer KL, Floyd CE, Harris CC, Coleman RE (1984). Improved SPECT quantitation using compensation for scattered photons. J Nucl Med.

[11] Ogawa K, Harata Y, Ichihara T, Kubo A, Hashimoto S (1991). A practical method for position-dependent Compton-scatter correction in single photon emission CT. IEEE Trans Med Imaging.

[12] Ljungberg M. The SIMIND Monte Carlo program Home Page. Available from:.

[13] Yeh DM, Chang PC, Pan LK (2013). The optimum Ga-67-citrate gamma camera imaging quality factors as first calculated and shown by the taguchi’s analysis. Hell J Nucl Med.

[14] Ljungberg M. The SIMIND Monte Carlo program Home Page.

[15] Rong X, Du Y, Ljungberg M, Rault E, Vandenberghe S, Frey EC (2012). Development and evaluation of an improved quantitative Y-90 bremsstrahlung SPECT method. Med Phys.

[16] Rong X, Frey EC (2013). A collimator optimization method for quantitative imaging: application to Y-90 bremsstrahlung SPECT. Med Phys.

[17] Toossi MB, Islamian JP, Momennezhad M, Ljungberg M, Naseri S (2010). SIMIND Monte Carlo simulation of a single photon emission CT. J Med Phys.

[18] Roshan HR, Mahmoudian B, Gharepapagh E, Azarm A, Islamian JP (2016). Collimator and energy window optimization for 90Y bremsstrahlung SPECT imaging: A SIMIND Monte Carlo stud. Appl Radia Isot.

[19] Lee YS, Kim JS, Kim KM, Lim SM, Kim HJ (2015;15). Determination of energy windows for the triple energy window scatter correction method in I-131 on a Siemens SYMBIA gamma camera: a GATE simulation study. J Inst.

[20] Chun SY, Fessler JA, Dewaraja YK (2013). Correction for Collimator-Detector Response in SPECT Using Point Spread Function Template. IEEE Trans Med Imaging.

[21] Bouzekraoui Y, Bentayeb F, Asmi H, Bonutti F (2019). Comparison of image quality of different radionuclides technetium-99m, samarium-153, and iodine-123. Indian J Nucl Med.

[22] Bouzekraoui Y, Bentayeb F, Asmi H, Bonutti F (2019). Energy window and contrast optimization for single-photon emission computed tomography bremsstrahlung imaging with yttrium-90. Indian J Nucl Med.

[23] Asmi H, Bentayeb F, Bouzekraoui Y, Bonutti F, Douama S (2020). Energy window and collimator optimization in lutetium-177 single photon emission computed tomography imaging using Monte Carlo simulation. Indian J Nucl Med.

[24] Asmi H, Bentayeb F, Bouzekraoui Y, Bonutti F (2019). Evaluation of acceptance angle in iodine-131 single photon emission computed tomography imaging with Monte Carlo simulation. Indian J Nucl Med.

[25] Bouzekraoui Y, Bentayeb F, Asmi H, Bonutti F (2019). Determination of Energy Windows for Triple Energy Window Scatter Correction Method in Gadolinium-159 Single Photon Emission Computed Tomography Using Monte Carlo Simulation. Iran J Med Phys.

